# Crystal structure of 4′-bromo-2′,5′-dimeth­oxy-2,5-dioxo-[1,1′-biphen­yl]-3,4-dicarbo­nitrile [BrHBQ(CN)_2_] benzene hemisolvate

**DOI:** 10.1107/S2056989016005120

**Published:** 2016-03-31

**Authors:** Joseph E. Meany, Deidra L. Gerlach, Elizabeth T. Papish, Stephen A. Woski

**Affiliations:** a Department of Chemistry, The University of Alabama, Box 870336, Tuscaloosa, AL, 35487, USA

**Keywords:** hemibi­quinone, mol­ecular rectifier, crystal structure

## Abstract

In the crystal of the title compound, C_16_H_9_BrN_2_O_4_·0.5C_6_H_6_, the mol­ecules stack in a centrosymmetric unit cell in a 2:1 stoichiometry with co-crystallized benzene solvent mol­ecules and inter­act *via* various weak inter­actions. This induces a geometry different from that predicted by theory, and is unique among the hemibi­quinones heretofore reported.

## Abstract 2

The geometry of the title hemibi­quinone is different from previous examples and may be correlated with the weak inter­actions in the crystal.

## Chemical context   

A new class of mol­ecules, dubbed hemibi­quinones (HBQs), has been developed and reported as potential mol­ecular rectifiers. Biphenyl derivatives have garnered great attention to themselves as conductors of electricity (Venkataraman *et al.*, 2006 ▸). Thus, control over the mol­ecular equilibrium geometry, and therefore the overlap of the π orbitals, allows for control over the governing electrical characteristics. The efficiency of conduction through a given mol­ecule is dependent on the torsion angle between adjacent electrophores.
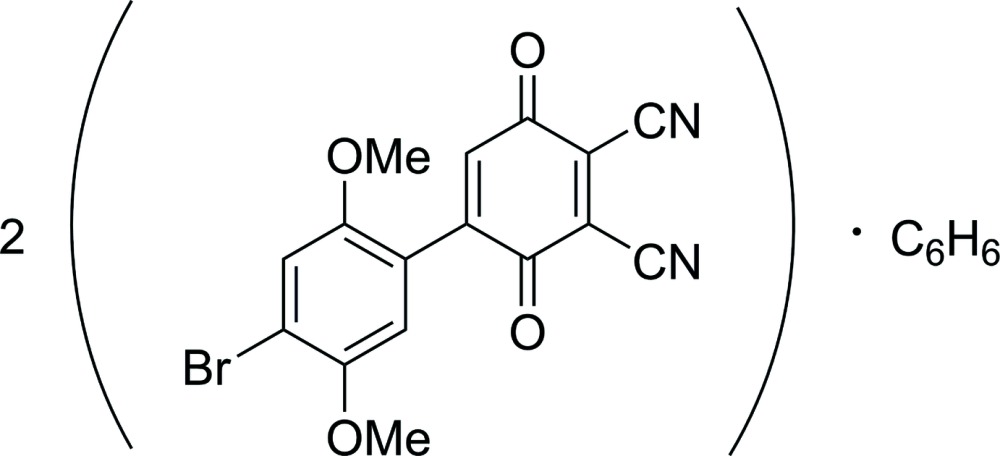


**Figure 1:**
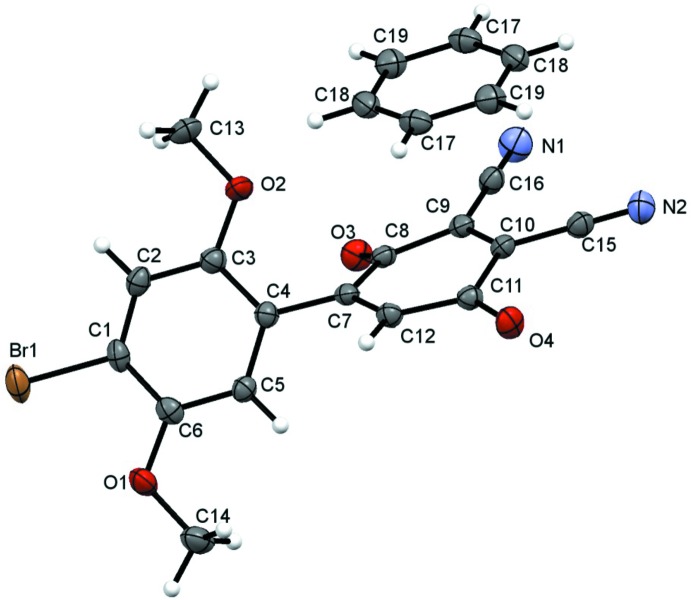
The mol­ecular structure of HBQ **1** showing the atom-numbering scheme. Ellipsoids are displayed at the 50% probability level. Hydrogen atoms are displayed as calculated.

The title hemibi­quinone (HBQ) mol­ecule, 4′-bromo-2′,5′-dimeth­oxy-2,5-dioxo-[1,1′-biphen­yl]-3,4-dicarbo­nitrile **1** (Fig. 1 ▸), has been isolated as a mol­ecule that will self-assemble on a gold surface as a potential unimolecular rectifier. HBQ **1** is predicted to act as a mol­ecular diode due to the linking of the electron-rich 4′-bromo-2′,5′-di­meth­oxy­benzene donor with an electron-poor 3,4-dicarbo­nitrile quinone acceptor. This follows the scheme outlined by Aviram & Ranter (1974 ▸), where an electron-rich donor and an electron-poor acceptor are covalently bonded through an isolating saturated bridge. In HBQs, the predicted dihedral twist away from coplanarity of the two rings would decrease orbital overlap and allow for partial isolation of the donor and acceptor moieties. We have developed a selective synthesis for this hemibi­quinone derivative that is scalable to gram qu­anti­ties.

## Structural commentary   

**Figure 2:**
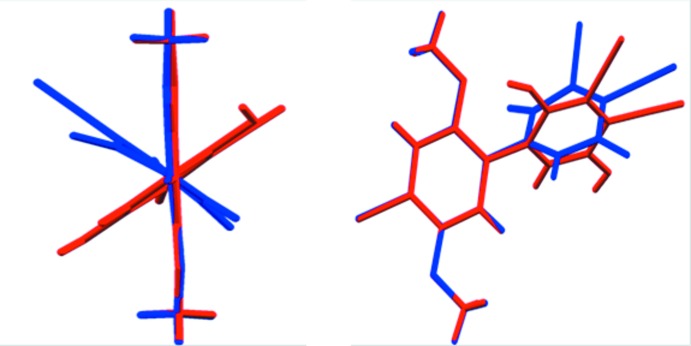
Mol­ecular overlay of **1** (blue) with the reduced precursor hydro­quinone (BrHBQH_2_(CN)_2_, red). Viewed along the plane of the di­meth­oxy­benzene ring and the C—C biphenyl bond (left), and parallel to the plane of the di­meth­oxy­benzene ring (right). The overlay is meant to show the divergent geometry between the precursor and the title mol­ecule, based on the different solid-state inter­actions.

As in the other reported HBQ mol­ecules (Meany *et al.*, 2015 ▸), we seek to use and compare the inter-ring torsion angles in the crystals as a guide against gas-phase calculated values. However, crystallized benzene solvent mol­ecules in the crystal structure prevent us from drawing direct conclusions about the geometry. Packing effects distort the biphenyl mol­ecule out of plane in the opposite direction as the hydro­quinone starting material [BrHBQH_2_(CN)_2_], Fig. 2 ▸. The C—C biphenyl bond [1.482 (2) Å] in **1** is comparable to that in the hydro­quinone [1.481 (2) Å]. In this mol­ecule, the C5—C4*—*C7—C8 torsion is 125.3 (2)°, compared to the hydro­quinone torsion angle of −126.50°, (Meany, 2015 ▸). DFT (B3LYP-DGDZVP) calculations performed on the target mol­ecule in the gas phase predict an angle of −39.71°. This significant discrepancy is due to packing inter­actions in the solid phase, especially from benzene. Finally, the quinone ring is slightly buckled, likely due to supra­molecular packing effects.

As in other structures, the meth­oxy groups are aligned nearly in-plane with the benzene ring, C2—C3—O2—C13 being bent out of plane by 2.9 (3)° and C5—C6—O1—C14 bent out of plane by −7.0 (3)°. The meth­oxy group bond angle C3—O2—C13 is measured at 117.4 (1)°, and C6—O1—C14 is measured at 117.3 (1)°. The methyl portions of each of these groups point away from the sterically restricting groups *ortho* to these positions, typical for this class.

Continuing the trend from the hydro­quinone, the C9—C10 bond in **1** [1.346 (2) Å] is shorter than than the corresponding hydro­quinone C9—C10 bond [1.408 (2) Å] and the C1—C6 bond [1.334 (6) Å] of BrHBQBr (Meany, 2015 ▸). The stronger polarization of **1** due to the di­cyano­quinone relative to the starting materials weakens the bond through repulsive effects. The Br1—C1 bond is slightly longer in **1** [1.893 (2) Å] compared to the same bond in the hydro­quinone precursor [1.885 (1) Å], but is still shorter than that of the starting material BrHBQBr [1.898 (4) Å; Meany, 2015 ▸]. The calculated dipole moment (B3LYP-DGDZVP) of BrHBQH_2_(CN)_2_ is only 6.17 D, compared to a dipole moment of 7.78 D for compound **1**.

## Supra­molecular features   

The mol­ecule packs in space group *P* 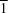 with two HBQ mol­ecules and one solvent mol­ecule, the latter completed by a crystallographic inversion centre. The mol­ecules align anti­parallel to one another in the unit cell, primarily along the *c* crystallographic axis. The quinone rings are mostly parallel to the *ac* plane and sandwich, in a 2:1 ratio, a benzene solvent mol­ecule. The plane of the di­meth­oxy­benzene ring aligns with the diagonal of the *ab* plane.

**Figure 3:**
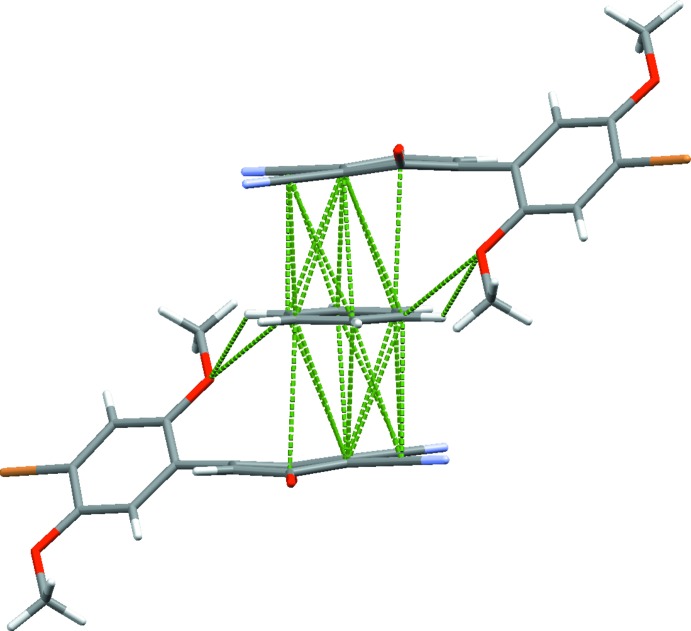
Packing diagram showing the off-center π–π short contacts of **1** with the inter­calated solvent benzene. Weak hydrogen bonds from O2 project closely to the benzene protons.

Analysis of the short contacts shows an off-center donor–acceptor-type π–π inter­action between benzene and the HBQ mol­ecule. Fig3. 3 ▸ and 4 ▸ show the great extent of π-overlap between benzene and HBQ **1**. It is readily apparent that the benzene ring, rather than being centered between the quinone rings exactly, is actually slightly off-center. Instead, the electron density of the benzene is centered over the slightly electropositive C9—C10 bond.

**Figure 5:**
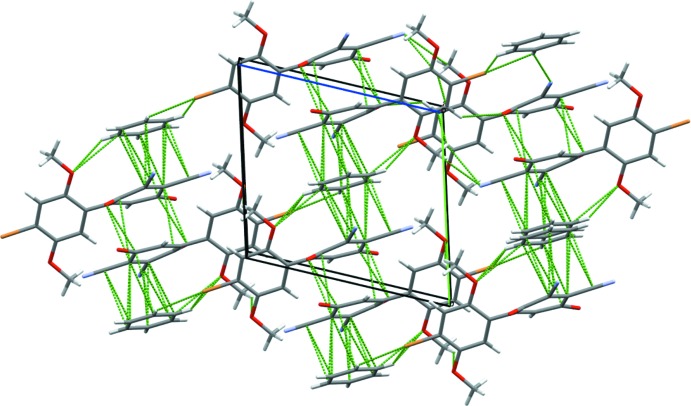
Packing diagram showing the *b* directional π-stacks and the weak *c* directional halogen bonds. Viewed along the *a* axis.

Each HBQ mol­ecule inter­acts with a total of three benzene mol­ecules by short contacts. As mentioned above, one mol­ecule of benzene is sandwiched between two quinone rings. Additionally, the 3-substituted nitrile group accepts a C—H⋯N hydrogen bond from a solvent mol­ecule (H⋯N = 2.81 Å, C—H⋯N = 147°). The third benzene mol­ecule exhibits short contacts to the 4′-bromine atom on the opposite end of the mol­ecule, where H17 and H18 link to Br1 almost symmetrically (H17⋯Br1 = 3.05 Å, C17—H17⋯Br1 = 127°; H18⋯Br1 = 3.04 Å, C18—H178⋯Br1 = 128°). Since the benzene mol­ecule π-stacks parallel to the quinones, the benzene mol­ecule is oriented in the same direction relative to the di­meth­oxy­benzene ring. Although, in previous HBQ crystals the 4 and 4′ groups show evidence of inter­molecular halogen bonding, due to the excess electron density around the aryl bromine atoms and the nitrile groups, an attractive inter­action is not possible, rather a slightly repulsive inter­action is favored. Instead, the protons on C17 and C18 bifurcate to Br1 as an acceptor, forming slightly asymmetric hydrogen bonds between the di­meth­oxy­benzene ring and the benzene solvent mol­ecule. As discussed above, the quinone carbonyl groups are deflected from perfect planarity. In previous structures, meth­oxy oxygen atoms tended to deflect the carbonyl groups through repulsive effects. However, this structure contains some attractive inter­molecular hydrogen bonding character, including the C14—H14*B*⋯O4 contact, which is a moderate inter­action at 2.57 Å and a bond angle of 157°. A second weaker inter­action occurs between the C5—H5 di­meth­oxy­benzene grouping and O4 (2.64 Å and 134°). Projection of the O4 carbonyl atom to a neighboring quinoid proton H12 is also evident at a bond length of 2.65 Å and a C12—H12⋯O4 angle of 147°. There is a fourth and weakest inter­action with O4, *viz*. C16≡N1⋯O4 with an N1⋯O4 bond length of 3.159 (2) Å and a bond angle of 129.91 (1)°. Two contacts are made with O3: a C2—H2⋯O3 (2.65 Å and 142°) bond and weak π-contacts [C10⋯O3 and C11⋯O3 = 3.251 (2) and 3.187 (2) Å, respectively]. Additionally, there is a short contact between C10 and C8, at 3.486 (3) Å. The N2 nitrile atom possibly accepts a very weak inter­action from the meth­oxy C13 and H13*A* pair (2.82 Å and 97°). There is a long C13⋯N2 [(3.101 (3) Å] inter­action as well. Even longer than those inter­actions, H13 also has a weak H⋯π inter­action with the di­meth­oxy­benzene ring on an adjacent mol­ecule (H⋯π = 2.88 Å). The packing is shown in Fig. 5 ▸.

## Synthesis and crystallization   

4′-Bromo-2,5-dihy­droxy-2′,5′-dimeth­oxy-[1,1′-biphen­yl]-3,4-dicarbo­nitrile (0.126 g, 0.337 mmol) was suspended in a mixture of 100 mL of H_2_O and 100 mL of benzene. FeCl_3_ (0.340 g, 2.09 mmol) was added in one portion. The resulting mixture was capped and stirred overnight. The resulting phases were separated, and the organic phase was washed with water and dried over anhydrous Na_2_SO_4_. Evaporation of the solvent produced a crude product. The pure product was precipitated from a chloro­form solution by addition of hexane, yielding 0.0460 g (36.7%). Black, block-shaped crystals of **1** were grown from chloro­form solution with residual benzene at 296 K. ^1^H NMR (360 MHz, CDCl_3_) δ = 7.22 (*s*, 1H, ArH), 7.12 (*s*, 1H, ArH), 6.71 (*s*, 1H, ArH), 3.87 (*s*, 3H, OCH_3_), 3.76 (*s*, 3H, OCH_3_).

## Refinement   

H atoms attached to carbon atoms were positioned geometrically and constrained to ride on their parent atoms, with C—H-bond distances of 0.95 Å for aromatic C—H, 1.00, 0.99 and 0.98 Å for aliphatic CH_3_, 0.88 Å. Methyl H atoms were allowed to rotate but not to tip to best fit the experimental electron density. *U*_iso_(H) values were set to a multiple of *U*_eq_(C) with 1.5 for CH_3_. Crystal data, data collection and structure refinement details are summarized in Table 1 ▸.

## Supplementary Material

Crystal structure: contains datablock(s) I. DOI: 10.1107/S2056989016005120/hb7567sup1.cif

Structure factors: contains datablock(s) I. DOI: 10.1107/S2056989016005120/hb7567Isup2.hkl

CCDC reference: 1470649

Additional supporting information:  crystallographic information; 3D view; checkCIF report

## Figures and Tables

**Figure 4:**
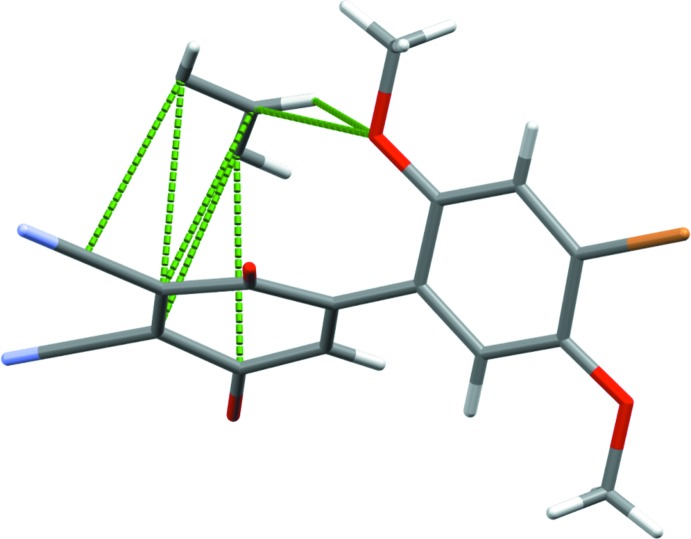
Packing diagram showing the off-center π–π short contacts of **1** with the inter­calated solvent benzene as the asymmetric unit to only one HBQ. Weak hydrogen bonds from O2 project close to the benzene protons.

**Table 1:** Experimental details

Crystal data	
Chemical formula	C_16_H_9_BrN_2_O_4_·0.5C_6_H_6_
*M*_r_	412.22
Crystal system, space group	Triclinic, *P* 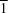
Temperature (K)	173
*a*, *b*, *c* (Å)	8.0427 (17), 10.343 (2), 11.007 (2)
α, β, γ (°)	104.469 (2), 95.120 (2), 102.851 (2)
*V* (Å^3^)	854.1 (3)
*Z*	2
Radiation type	Mo *K*α
μ (mm^−1^)	2.43
Crystal size (mm)	0.21 × 0.11 × 0.06

Data collection	
Diffractometer	Bruker AXS *SMART* APEXII CCD
Absorption correction	Multi-scan (*SADABS*; Bruker, 2001 ▸)
*T*_min_, *T*_max_	0.600, 0.746
No. of measured, independent and observed [*I* > 2σ(*I*)] reflections	11856, 3957, 3382
*R*_int_	0.022
(sin θ/λ)_max_ (Å^−1^)	0.653

Refinement	
*R*[*F* ^2^ > 2σ(*F* ^2^)], *wR*(*F* ^2^), *S*	0.028, 0.068, 1.06
No. of reflections	3957
No. of parameters	237
H-atom treatment	H-atom parameters constrained
Δρ_max_, Δρ_min_ (e Å^−3^)	0.40, −0.35

Computer programs: *APEX2* and *SAINT* (Bruker, 2012 ▸), *SHELXT* (Sheldrick, 2015*a* ▸), *SHELXL2014/7* (Sheldrick, 2015*b* ▸) *SHELXLE* (Hübschle *et al.*, 2011 ▸), *Mercury* (Macrae *et al.*, 2006 ▸) and *SHELXTL* (Sheldrick, 2008 ▸).

## supplementary crystallographic information

### Crystal data

**Table:**

C_16_H_9_BrN_2_O_4_·0.5C_6_H_6_	*Z* = 2
*M**_r_* = 412.22	*F*(000) = 414
Triclinic, *P*1	*D*_x_ = 1.603 Mg m^−^^3^
*a* = 8.0427 (17) Å	Mo *K*α radiation, λ = 0.71073 Å
*b* = 10.343 (2) Å	Cell parameters from 5182 reflections
*c* = 11.007 (2) Å	θ = 2.9–27.4°
α = 104.469 (2)°	µ = 2.43 mm^−^^1^
β = 95.120 (2)°	*T* = 173 K
γ = 102.851 (2)°	Block, black
*V* = 854.1 (3) Å^3^	0.21 × 0.11 × 0.06 mm

### Data collection

**Table:**

Bruker AXS SMART APEXII CCD diffractometer	3382 reflections with *I* > 2σ(*I*)
Radiation source: sealed X-ray tube	*R*_int_ = 0.022
phi and ω scans	θ_max_ = 27.6°, θ_min_ = 2.9°
Absorption correction: multi-scan (*SADABS*; Bruker, 2001)	*h* = −10→10
*T*_min_ = 0.600, *T*_max_ = 0.746	*k* = −13→13
11856 measured reflections	*l* = −14→14
3957 independent reflections	

### Refinement

**Table:**

Refinement on *F*^2^	Primary atom site location: structure-invariant direct methods
Least-squares matrix: full	Secondary atom site location: difference Fourier map
*R*[*F*^2^ > 2σ(*F*^2^)] = 0.028	Hydrogen site location: inferred from neighbouring sites
*wR*(*F*^2^) = 0.068	H-atom parameters constrained
*S* = 1.06	*w* = 1/[σ^2^(*F*_o_^2^) + (0.0307*P*)^2^ + 0.3076*P*] where *P* = (*F*_o_^2^ + 2*F*_c_^2^)/3
3957 reflections	(Δ/σ)_max_ < 0.001
237 parameters	Δρ_max_ = 0.40 e Å^−^^3^
0 restraints	Δρ_min_ = −0.35 e Å^−^^3^

### Special details

**Table:**

Geometry. All esds (except the esd in the dihedral angle between two l.s. planes) are estimated using the full covariance matrix. The cell esds are taken into account individually in the estimation of esds in distances, angles and torsion angles; correlations between esds in cell parameters are only used when they are defined by crystal symmetry. An approximate (isotropic) treatment of cell esds is used for estimating esds involving l.s. planes.

### Fractional atomic coordinates and isotropic or equivalent isotropic displacement parameters (Å^2^)

**Table:**

	*x*	*y*	*z*	*U*_iso_*/*U*_eq_	
C1	0.1145 (2)	−0.15670 (19)	−0.05223 (15)	0.0248 (4)	
C2	0.2309 (2)	−0.02953 (19)	−0.02559 (16)	0.0249 (4)	
H2	0.2677	0.0071	−0.0925	0.030*	
C3	0.2939 (2)	0.04468 (18)	0.10043 (16)	0.0240 (4)	
C4	0.2382 (2)	−0.01088 (18)	0.19817 (15)	0.0216 (3)	
C5	0.1153 (2)	−0.13776 (18)	0.16872 (15)	0.0219 (3)	
H5	0.0746	−0.1731	0.2353	0.026*	
C6	0.0521 (2)	−0.21285 (18)	0.04302 (16)	0.0235 (4)	
C7	0.3088 (2)	0.06338 (17)	0.33278 (15)	0.0207 (3)	
C8	0.5003 (2)	0.10333 (17)	0.36973 (15)	0.0207 (3)	
C9	0.5736 (2)	0.19577 (17)	0.50147 (15)	0.0207 (3)	
C10	0.4713 (2)	0.21563 (17)	0.59112 (15)	0.0212 (3)	
C11	0.2798 (2)	0.15131 (17)	0.55889 (16)	0.0215 (3)	
C12	0.2090 (2)	0.08304 (18)	0.42447 (16)	0.0224 (3)	
H12	0.0872	0.0513	0.4011	0.027*	
C13	0.4568 (3)	0.2362 (2)	0.04085 (19)	0.0327 (4)	
H13A	0.5171	0.1797	−0.0155	0.049*	
H13B	0.5338	0.3282	0.0801	0.049*	
H13C	0.3540	0.2445	−0.0084	0.049*	
C14	−0.1132 (3)	−0.4035 (2)	0.10228 (19)	0.0356 (4)	
H14A	−0.1932	−0.4943	0.0630	0.053*	
H14B	−0.1695	−0.3463	0.1616	0.053*	
H14C	−0.0097	−0.4148	0.1483	0.053*	
C15	0.5379 (2)	0.29235 (19)	0.72153 (16)	0.0260 (4)	
C16	0.7587 (2)	0.24907 (19)	0.52856 (16)	0.0257 (4)	
C17	0.3212 (2)	0.45790 (19)	0.48189 (19)	0.0298 (4)	
H17	0.1988	0.4292	0.4699	0.036*	
C18	0.4079 (3)	0.43078 (19)	0.37931 (18)	0.0301 (4)	
H18	0.3454	0.3836	0.2969	0.036*	
C19	0.5876 (3)	0.4731 (2)	0.39738 (18)	0.0306 (4)	
H19	0.6478	0.4547	0.3271	0.037*	
N1	0.9050 (2)	0.2909 (2)	0.55007 (17)	0.0393 (4)	
N2	0.5915 (2)	0.35262 (19)	0.82435 (15)	0.0399 (4)	
O1	−0.06536 (16)	−0.33788 (13)	0.00550 (12)	0.0311 (3)	
O2	0.40645 (18)	0.17220 (13)	0.13801 (12)	0.0327 (3)	
O3	0.59685 (17)	0.05565 (14)	0.30356 (12)	0.0308 (3)	
O4	0.19158 (16)	0.15713 (14)	0.64297 (12)	0.0303 (3)	
Br1	0.04198 (3)	−0.26003 (2)	−0.22428 (2)	0.03965 (8)	

### Atomic displacement parameters (Å^2^)

**Table:**

	*U*^11^	*U*^22^	*U*^33^	*U*^12^	*U*^13^	*U*^23^
C1	0.0214 (8)	0.0332 (10)	0.0170 (8)	0.0076 (7)	0.0021 (6)	0.0015 (7)
C2	0.0256 (9)	0.0318 (10)	0.0200 (8)	0.0081 (8)	0.0074 (7)	0.0098 (7)
C3	0.0254 (9)	0.0231 (9)	0.0231 (8)	0.0045 (7)	0.0069 (7)	0.0062 (7)
C4	0.0235 (8)	0.0217 (9)	0.0190 (8)	0.0055 (7)	0.0057 (6)	0.0042 (6)
C5	0.0225 (8)	0.0242 (9)	0.0194 (8)	0.0058 (7)	0.0062 (6)	0.0059 (7)
C6	0.0180 (8)	0.0256 (9)	0.0248 (8)	0.0044 (7)	0.0040 (6)	0.0035 (7)
C7	0.0242 (8)	0.0165 (8)	0.0211 (8)	0.0021 (7)	0.0053 (6)	0.0065 (6)
C8	0.0230 (8)	0.0193 (8)	0.0212 (8)	0.0033 (7)	0.0081 (6)	0.0085 (6)
C9	0.0208 (8)	0.0199 (8)	0.0229 (8)	0.0041 (7)	0.0039 (6)	0.0093 (7)
C10	0.0239 (8)	0.0208 (9)	0.0195 (8)	0.0034 (7)	0.0048 (6)	0.0080 (6)
C11	0.0223 (8)	0.0208 (8)	0.0220 (8)	0.0048 (7)	0.0068 (6)	0.0065 (6)
C12	0.0205 (8)	0.0226 (9)	0.0218 (8)	0.0015 (7)	0.0047 (6)	0.0051 (7)
C13	0.0354 (10)	0.0330 (10)	0.0332 (10)	0.0033 (9)	0.0115 (8)	0.0182 (8)
C14	0.0371 (11)	0.0271 (10)	0.0350 (10)	−0.0038 (9)	0.0107 (8)	0.0037 (8)
C15	0.0250 (9)	0.0292 (10)	0.0240 (9)	0.0031 (8)	0.0054 (7)	0.0108 (7)
C16	0.0256 (9)	0.0307 (10)	0.0227 (8)	0.0059 (8)	0.0056 (7)	0.0114 (7)
C17	0.0237 (9)	0.0242 (9)	0.0399 (10)	0.0022 (8)	0.0026 (8)	0.0105 (8)
C18	0.0329 (10)	0.0237 (9)	0.0293 (9)	0.0030 (8)	−0.0015 (8)	0.0053 (7)
C19	0.0329 (10)	0.0282 (10)	0.0322 (10)	0.0088 (8)	0.0098 (8)	0.0083 (8)
N1	0.0243 (9)	0.0536 (12)	0.0407 (10)	0.0062 (8)	0.0046 (7)	0.0181 (9)
N2	0.0457 (10)	0.0428 (10)	0.0235 (8)	−0.0013 (8)	0.0018 (7)	0.0077 (7)
O1	0.0283 (7)	0.0286 (7)	0.0267 (6)	−0.0046 (6)	0.0027 (5)	0.0013 (5)
O2	0.0445 (8)	0.0254 (7)	0.0232 (6)	−0.0044 (6)	0.0095 (6)	0.0078 (5)
O3	0.0284 (7)	0.0338 (7)	0.0307 (7)	0.0086 (6)	0.0142 (5)	0.0058 (6)
O4	0.0271 (7)	0.0380 (8)	0.0241 (6)	0.0055 (6)	0.0113 (5)	0.0054 (5)
Br1	0.03537 (12)	0.05225 (15)	0.01873 (10)	−0.00172 (9)	0.00001 (7)	0.00002 (8)

### Geometric parameters (Å, º)

**Table:**

C1—C2	1.379 (3)	C11—O4	1.213 (2)
C1—C6	1.399 (2)	C11—C12	1.470 (2)
C1—Br1	1.8928 (17)	C12—H12	0.9500
C2—C3	1.393 (2)	C13—O2	1.434 (2)
C2—H2	0.9500	C13—H13A	0.9800
C3—O2	1.364 (2)	C13—H13B	0.9800
C3—C4	1.403 (2)	C13—H13C	0.9800
C4—C5	1.399 (2)	C14—O1	1.436 (2)
C4—C7	1.482 (2)	C14—H14A	0.9800
C5—C6	1.392 (2)	C14—H14B	0.9800
C5—H5	0.9500	C14—H14C	0.9800
C6—O1	1.362 (2)	C15—N2	1.138 (2)
C7—C12	1.349 (2)	C16—N1	1.141 (2)
C7—C8	1.493 (2)	C17—C18	1.382 (3)
C8—O3	1.212 (2)	C17—C19^i^	1.390 (3)
C8—C9	1.506 (2)	C17—H17	0.9500
C9—C10	1.346 (2)	C18—C19	1.394 (3)
C9—C16	1.443 (2)	C18—H18	0.9500
C10—C15	1.443 (2)	C19—C17^i^	1.390 (3)
C10—C11	1.506 (2)	C19—H19	0.9500
			
C2—C1—C6	122.44 (15)	O4—C11—C10	119.64 (15)
C2—C1—Br1	118.35 (13)	C12—C11—C10	117.32 (14)
C6—C1—Br1	119.19 (13)	C7—C12—C11	123.04 (15)
C1—C2—C3	119.35 (15)	C7—C12—H12	118.5
C1—C2—H2	120.3	C11—C12—H12	118.5
C3—C2—H2	120.3	O2—C13—H13A	109.5
O2—C3—C2	124.46 (15)	O2—C13—H13B	109.5
O2—C3—C4	115.94 (15)	H13A—C13—H13B	109.5
C2—C3—C4	119.59 (16)	O2—C13—H13C	109.5
C5—C4—C3	119.93 (15)	H13A—C13—H13C	109.5
C5—C4—C7	119.47 (14)	H13B—C13—H13C	109.5
C3—C4—C7	120.60 (15)	O1—C14—H14A	109.5
C6—C5—C4	120.83 (15)	O1—C14—H14B	109.5
C6—C5—H5	119.6	H14A—C14—H14B	109.5
C4—C5—H5	119.6	O1—C14—H14C	109.5
O1—C6—C5	124.89 (15)	H14A—C14—H14C	109.5
O1—C6—C1	117.32 (15)	H14B—C14—H14C	109.5
C5—C6—C1	117.79 (16)	N2—C15—C10	179.5 (2)
C12—C7—C4	123.16 (15)	N1—C16—C9	179.8 (2)
C12—C7—C8	118.87 (15)	C18—C17—C19^i^	120.29 (17)
C4—C7—C8	117.58 (14)	C18—C17—H17	119.9
O3—C8—C7	123.21 (15)	C19^i^—C17—H17	119.9
O3—C8—C9	118.80 (15)	C17—C18—C19	119.66 (17)
C7—C8—C9	117.66 (14)	C17—C18—H18	120.2
C10—C9—C16	122.54 (15)	C19—C18—H18	120.2
C10—C9—C8	120.68 (15)	C17^i^—C19—C18	120.05 (18)
C16—C9—C8	116.49 (14)	C17^i^—C19—H19	120.0
C9—C10—C15	122.68 (15)	C18—C19—H19	120.0
C9—C10—C11	120.23 (15)	C6—O1—C14	117.33 (14)
C15—C10—C11	117.03 (14)	C3—O2—C13	117.42 (14)
O4—C11—C12	123.04 (16)		
			
C6—C1—C2—C3	2.2 (3)	C4—C7—C8—C9	171.61 (14)
Br1—C1—C2—C3	−176.22 (13)	O3—C8—C9—C10	−158.87 (16)
C1—C2—C3—O2	−178.67 (17)	C7—C8—C9—C10	14.8 (2)
C1—C2—C3—C4	0.0 (3)	O3—C8—C9—C16	15.1 (2)
O2—C3—C4—C5	176.45 (16)	C7—C8—C9—C16	−171.25 (14)
C2—C3—C4—C5	−2.3 (3)	C16—C9—C10—C15	0.3 (3)
O2—C3—C4—C7	−3.8 (2)	C8—C9—C10—C15	173.92 (15)
C2—C3—C4—C7	177.44 (16)	C16—C9—C10—C11	−176.80 (15)
C3—C4—C5—C6	2.5 (3)	C8—C9—C10—C11	−3.2 (2)
C7—C4—C5—C6	−177.20 (15)	C9—C10—C11—O4	171.98 (16)
C4—C5—C6—O1	178.97 (16)	C15—C10—C11—O4	−5.3 (2)
C4—C5—C6—C1	−0.4 (3)	C9—C10—C11—C12	−7.8 (2)
C2—C1—C6—O1	178.57 (16)	C15—C10—C11—C12	174.96 (15)
Br1—C1—C6—O1	−3.0 (2)	C4—C7—C12—C11	177.18 (15)
C2—C1—C6—C5	−2.0 (3)	C8—C7—C12—C11	4.5 (2)
Br1—C1—C6—C5	176.44 (12)	O4—C11—C12—C7	−172.59 (17)
C5—C4—C7—C12	−47.4 (2)	C10—C11—C12—C7	7.1 (2)
C3—C4—C7—C12	132.83 (18)	C19^i^—C17—C18—C19	0.0 (3)
C5—C4—C7—C8	125.34 (17)	C17—C18—C19—C17^i^	0.0 (3)
C3—C4—C7—C8	−54.4 (2)	C5—C6—O1—C14	−7.0 (3)
C12—C7—C8—O3	158.02 (17)	C1—C6—O1—C14	172.39 (16)
C4—C7—C8—O3	−15.1 (2)	C2—C3—O2—C13	2.9 (3)
C12—C7—C8—C9	−15.3 (2)	C4—C3—O2—C13	−175.80 (16)

Symmetry code: (i) −*x*+1, −*y*+1, −*z*+1.
